# Metformin Protects Rat Hepatocytes against Bile Acid-Induced Apoptosis

**DOI:** 10.1371/journal.pone.0071773

**Published:** 2013-08-12

**Authors:** Titia E. Woudenberg-Vrenken, Laura Conde de la Rosa, Manon Buist-Homan, Klaas Nico Faber, Han Moshage

**Affiliations:** Department of Gastroenterology and Hepatology, University Medical Center Groningen, University of Groningen, Groningen, The Netherlands; University of Nebraska Medical Center, United States of America

## Abstract

**Background:**

Metformin is used in the treatment of Diabetes Mellitus type II and improves liver function in patients with non-alcoholic fatty liver disease (NAFLD). Metformin activates AMP-activated protein kinase (AMPK), the cellular energy sensor that is sensitive to changes in the AMP/ATP-ratio. AMPK is an inhibitor of mammalian target of rapamycin (mTOR). Both AMPK and mTOR are able to modulate cell death.

**Aim:**

To evaluate the effects of metformin on hepatocyte cell death.

**Methods:**

Apoptotic cell death was induced in primary rat hepatocytes using either the bile acid glycochenodeoxycholic acid (GCDCA) or TNFα in combination with actinomycin D (actD). AMPK, mTOR and phosphoinositide-3 kinase (PI3K)/Akt were inhibited using pharmacological inhibitors. Apoptosis and necrosis were quantified by caspase activation, acridine orange staining and Sytox green staining respectively.

**Results:**

Metformin dose-dependently reduces GCDCA-induced apoptosis, even when added 2 hours after GCDCA, without increasing necrotic cell death. Metformin does not protect against TNFα/ActD-induced apoptosis. The protective effect of metformin is dependent on an intact PI3-kinase/Akt pathway, but does not require AMPK/mTOR-signaling. Metformin does not inhibit NF-κB activation.

**Conclusion:**

Metformin protects against bile acid-induced apoptosis and could be considered in the treatment of chronic liver diseases accompanied by inflammation.

## Introduction

Metformin is a drug primarily used in the treatment of Diabetes Mellitus type II where it suppresses glucose production by the liver. Recently, metformin was shown to have beneficial effects in patients with (non-alcoholic) fatty liver diseases (NAFLD) and poly-cystic ovarian syndrome (PCOS) [1], [2]. In patients and in vivo models of non-alcoholic steatohepatitis (NASH), metformin reduced leptin secretion and aminotransferase levels and decreased liver size. Moreover, metformin treatment improved hepatocyte viability in fatty livers [3]–[8]. In addition, metformin protected hepatocytes from cell death induced by saturated fatty acids [9].

Metformin is known to stimulate AMP-activated protein kinase (AMPK) activity both in whole liver, primary hepatocytes, and a hepatoma cell line [10]–[12]. Among the 5 members of the AMPK family are AMPK-α1 and -α2 that are activated by metformin [10], [13]. AMPK consists of a catalytic α subunit and two regulatory subunits (β, γ; [10], [14]. AMPK is involved in insulin signaling, energy homeostasis, and becomes activated upon a rise in cellular AMP concentration or changes in the AMP/ATP-ratio. Furthermore, AMPK can be activated by stimuli that do not affect the AMP/ATP-ratio, like hyperosmotic stress, hypoxia, oxidative stress or pharmacological compounds [12], [14]–[20]. AMPK activity is dependent on the phosphorylation of Thr172 in the α subunit [21].

Activation of AMPK using the cell permeable adenosine analogue 5-aminoimidazole-4-carboxamide 1-β-D-ribofuranoside (AICAR) was shown to be pro-apoptotic, via activation of JNK and caspase-3 in liver cells [22]. Also, in a rat hepatoma cell line AMPK activity stimulated apoptosis, and in pancreatic β-cells both metformin and AICAR induced apoptosis. In contrast, AMPK activation reduced apoptosis in astrocytes and endothelial cells [23]. Moreover, in DLD-1 cells, Ark5, another AMPK family member, was protective against Fas-mediated cell death. Ark5 directly inhibited one of the effector caspases, caspase-6, and Ark5 activity was shown to be controlled by Akt, a key regulator in survival signaling [11], [15], [24]–[27]. In whole liver, AMPK activity represses signaling via mammalian target of rapamycin (mTOR), a downstream target of Akt and phosphoinositide-3 kinase (PI3K). mTOR is a key player in transcription, translation, cytoskeletal arrangement, and protein degradation [14], [16], [26], [28]–[33]. Akt was found to suppress apoptosis in various cell types, including liver cells. In a rat hepatoma cell line, constitutive activation of PI3K blocks GCDCA-induced apoptosis. In primary rat hepatocytes, the protection of tauroursodeoxycholic acid (TUDCA) against GCDCA-induced apoptosis was abolished when the PI3K/Akt survival pathway was inhibited [34]–[39]. Several important survival pathways next to PI3K/Akt are present in hepatocytes, like the transcription factor nuclear factor-κB (NF-κB) and the mitogen activated protein (MAP) kinases [40]. Activation of NF-κB leads to the induction of survival genes and subsequently inhibition of apoptosis. In cholestatic livers, NF-κB is activated, and reduces liver injury [41], and glycochenodeoxycholic acid (GCDCA)-induced apoptosis was reduced by NF-κB activation in primary rat hepatocytes *in vitro* [42]. Furthermore, inhibition of both the PI3K pathway and one of the MAP-kinases p38 or ERK enhanced bile acid induced cell death in rat hepatocytes [43].

Considering the controversy about the role of metformin and its downstream target AMPK in apoptosis and the apparent beneficial effect of the drug in the treatment of NAFLD, we investigated the effect of metformin in two different models of hepatocyte damage. We studied an *in vitro* model of acute liver damage triggered by cytokines and a model of chronic liver disease induced by bile acids. We investigated whether metformin has effects on hepatocyte survival pathways and whether downstream targets of metformin modulate hepatocyte cell death. We describe a hepatoprotective action of metformin against bile acid-induced apoptosis that is independent of AMPK activation, but dependent on an intact PI3K/Akt signaling pathway.

## Materials and Methods

### Animals

Specified pathogen-free male Wistar rats (200–250 g) were purchased from Charles River Laboratories Inc (Wilmington, MA, USA). Rats were housed under standard laboratory conditions with free access to standard laboratory chow and water. Prior to isolation, rats were fasted overnight, and anesthetized using a combination of medetomidine/ketamine (i.p. injection). Experiments were approved by the Institutional Animal Care and Use Committee of the University of Groningen (IACUC-RuG) and were performed following their guidelines.

### Rat Hepatocyte Isolation

Hepatocytes were isolated from rats by a two step collagenase perfusion as described before [44]. Cell viability was greater than 85% as determined by Trypan Blue exclusion. After isolation, 112,500 cells per cm^2^ were plated on Vitrogen® (Cohesion Technologies Inc., Palo Alto, CA, USA) coated plates in William’s E medium (Invitrogen, Breda, The Netherlands) supplemented with 50 µg/mL gentamycin (Invitrogen) and penicillin-streptomycin-fungizone (Lonza, Verviers, Belgium). During the attachment period (4 hrs) 50 nmol/L dexamethasone (Dept. of Pharmacy, UMCG, Groningen, The Netherlands) and 5% fetal calf serum (Invitrogen) were added to the medium. Cells were cultured in a humidified incubator at 37°C and 5% CO_2_.

### Experimental Design

Experiments were started 4 hrs after isolation of hepatocytes. Monolayer cultures were exposed to 50 µmol/L GCDCA (Calbiochem, La Jolla, CA, USA) for 4 hrs or the indicated time period, or for 16 hrs to 20 ng/ml recombinant murine tumor necrosis factor α (mTNFα, R&D Systems, Abingdon, United Kingdom) in combination with actinomycin-D (ActD, Roche Diagnostics, Almere, The Netherlands, 200 ng/ml) or a cytokine mixture consisting of 20 ng/ml mTNFα, 10 ng/ml recombinant human interleukin-1β (hIL-1β, R&D Systems), 10 ng/ml recombinant rat interferon-γ (rIFNγ, R&D Systems.) and 10 µg/ml LPS (*Escherichia coli*, serotype 0127:B8, Sigma-Aldrich, St. Louis, MO, USA). Bile acid uptake was studied using the fluorescein labeled bile acid cholyl-lysyl-fluorescein (CLF, BD Biosciences, Breda, The Netherlands) 2 µmol/L for 1 hr. 1,1-dimethylbiguanide hydrochloride (metformin, Sigma-Aldrich) was used at a concentration range from 0.1–2 mmol/L. Signal transduction pathways were specifically blocked using 0.1 µmol/L of the AMPK inhibitor 5′-iodotubercidin (Calbiochem), 0.5 µmol/L of the mTOR inhibitor rapamycin (Calbiochem) or 50 µmol/L of the PI3K inhibitor LY 294002 (Calbiochem). All inhibitors were added 30 minutes prior to bile acids or cytokines. Each experimental condition was performed in triplicate wells. Each experiment was performed at least three times, using hepatocytes from different isolations.

Cells were harvested at the indicated time points after the addition of the apoptotic stimuli, and washed three times with ice cold phosphate buffered saline (PBS) before the addition of hypotonic cell lysis buffer (protein analysis, caspase-3 assay), 2-times concentrated sample buffer (Western blot analysis) or Tri-reagent (RNA isolation, Sigma-Aldrich) as described previously [43].

### Caspase Enzyme Activity Assays

Caspase-3 like activity was assayed as described previously [43]. The arbitrary fluorescence unit (AFU) was corrected for the amount of protein in the cell lysate. Caspase-6 like activity was assayed according to the manufacturer’s instructions (Biovision, Mountain View, CA, USA).

### Sytox Green and Acridine Orange Nuclear Staining

To determine necrotic cell death at the indicated time points, hepatocytes were incubated for 15 minutes with Sytox green (Invitrogen) nucleic acid stain. Sytox green can only enter cells with compromised plasma membranes, and cannot cross the membranes of viable cells or apoptotic bodies. Hepatocytes exposed to 5 mmol/L H_2_O_2_ (Merck Chemicals Ltd, Nottingham, UK) for 6 hrs served as positive control for necrosis. Apoptotic nuclei were visualized with acridine orange (Sigma-Aldrich) as described previously [44]. Fluorescent nuclei were visualized using an Olympus CKX41 microscope at 450–490 nm. Necrosis and apoptosis were quantified by counting fluorescent nuclei (necrotic or apoptotic cells) and the total number of cells in 3 randomly chosen high power fields.

### Western Blot Analysis

Western blot analysis of cell lysates was performed by SDS-PAGE followed by semi dry-blotting to transfer the proteins to Hybond ECL nitrocellulose membrane (Amersham Biosciences, Piscataway, NJ, USA). Ponceau S 0.1% w/v (Sigma-Aldrich) staining was used to ensure electrophoretic transfer. Activation of AMPK was detected using the polyclonal antibody against phosphorylated AMPKα (Thr 172, Invitrogen) at a dilution of 1∶500. Hepatocytes exposed to 250 µmol/L AICAR (Biomol Research Laboratories Inc, Plymouth Meeting, PA, USA) for 60 minutes served as positive control for phosphorylation and activation of AMPK. Akt activation was detected using the polyclonal antibody against phosphorylated Akt (Ser473, Cell Signaling Technology, Danvers, MA, USA) at a dilution of 1∶1000. After Western blot analysis, blots were stripped as described previously [44] and incubated with a monoclonal antibody against β-actin (Sigma-Aldrich, dilution 1∶10000) or GAPDH (Calbiochem, dilution 1∶50000).

The blots were analyzed in a ChemiDoc XRS system (Bio-Rad, Hercules, CA, USA). Protein band intensities were quantified by Quantity One software (Bio-Rad).

### RNA Isolation and Quantitative PCR

RNA isolation was performed as described previously [45]. RNA concentration was determined with the Ribogreen RNA quantitation reagent and kit (Invitrogen). Reverse transcription PCR (RT-PCR) was carried out on 2.5 µg of total RNA using random primers in a total volume of 50 µL using Moloney murine leukemia virus (M-MLV) reverse transcriptase system (Sigma-Aldrich) according to the manufacturer’s instruction.

Quantitative PCR was performed on 4 µL 20-times diluted complementary DNA in a final volume of 20 µL [45]. Fluorescence was measured using the ABI PRISM 7700 Sequence Detector version 1.7 software (Applied Biosystems, Nieuwerkerk a/d IJssel, The Netherlands) starting with 10 minutes at 95°C, followed by 40 cycles of 15 seconds at 95°C and 1 minute at 60°C. Details of primers and probes are listed in Table 1. Each sample was analyzed in duplicate. 18S mRNA levels were used as endogenous control.

**Table 1:** Sequences of primers and probes used for quantitative PCR analysis.

Rat 18S	Sense	5′-CGG CTA CCA CAT CCA AGG A-3′
	Antisense	5′-CCA ATT ACA GGG CCT CGA AA-3′
	Probe	5′FAM-CGC GCA AAT TAC CCA CTC CCG A-TAMRA3′
Rat iNOS	Sense	5′-GTG CTA ATG CGG AAG GTC ATG-3′
	Antisense	5′-CGA CTT TCC TGT CTC AGT AGC AAA-3′
	Probe	5′FAM-CCC GCG TCA GAG CCA CAG TCC T-TAMRA3′

### Statistical Analysis

All numerical results are reported as the mean of at least 3 independent experiments ± standard error of the mean. For each experiment, the results were analyzed using the Kruskal-Wallis test to verify the significance. If P<0.05 for the Kruskal-Wallis test, a Mann-Whitney U test was used to determine the significance of differences between experimental groups. A P-value smaller than 0.05 was considered to be statistically significant.

## Results

### Metformin Dose-dependently Reduces Bile Acid Induced Apoptosis

**Figure 1:**
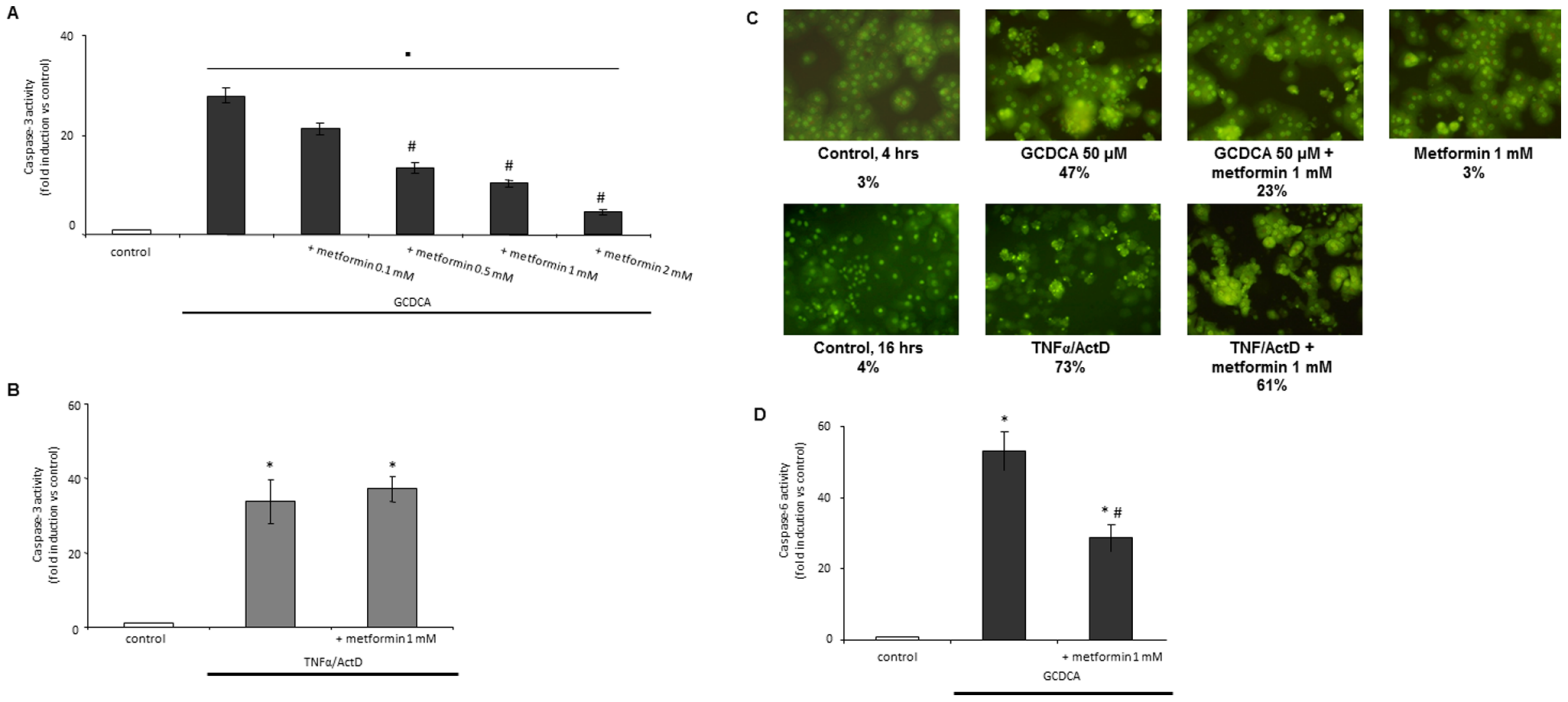
Metformin dose-dependently reduces bile acid-induced caspase-3 activity, but not TNFα/ActD-induced apoptosis. Primary rat hepatocytes were exposed to metformin (0.1–2 mmol/L) added 10 minutes prior to (A) GCDCA (50 µmol/L, 4 hrs) or (B) TNFα/ActD (20 ng/ml, 200 ng/ml, 16 hrs). Caspase-3 like activity is shown as fold induction compared to control values; control values were set at one. (C) Metformin prevents GCDCA (4 hrs) induced nuclear condensation as demonstrated by acridine orange staining, but has no effect on TNFα/ActD (16 hrs) induced nuclear condensation. Percentages represent condensed nuclei. Magnification 20X. (D) Metformin also reduces GCDCA-induced caspase-6 activity. Caspase-6 like activity was measured in primary rat hepatocytes exposed to GCDCA (50 µmol/L, 4 hrs) and/or metformin (1 mmol/L) and is shown as fold induction compared to control values; control values were set at one. N = 4 for each experiment. Statistical analysis: * p<0.05 or ▪ p<0.01 compared to control. # p<0.05 compared to GCDCA-treated cells.

To examine a potential hepatoprotective effect of metformin, two different models for apoptosis were used: bile acid (GCDCA)-induced apoptosis and TNFα/ActD-induced apoptosis. GCDCA-induced caspase-3 activity peaks at 4 hrs [43], while for TNFα/ActD-induced apoptosis, caspase-3 activity is maximal between 12–16 hrs (data not shown). Therefore, these time points were chosen to study the beneficial effect of metformin. Metformin inhibited GCDCA-induced caspase-3 activity dose-dependently from 24% inhibition at 0.1 mmol/L to an almost complete block (84% inhibition) at a concentration of 2 mmol/L (Fig. 1A). In contrast, 1 mmol/L metformin, a concentration that blocks GCDCA-induced caspase-3 activity for 63%, had no effect on TNFα/ActD-induced caspase-3 activity (Fig. 1B). Metformin (0.1–2 mmol/L) alone did not induce caspase-3 activity. However, higher concentrations (5 mmol/L and higher) were toxic to hepatocytes (data not shown). Acridine orange staining confirmed the results obtained with the caspase-3 assay. Condensed nuclei are visible in 47% of GCDCA-treated hepatocytes (Fig. 1C), whereas in combination with metformin this number of apoptotic cells is reduced by half to 23%. However, the TNFα/ActD-induced nuclear condensation is hardly affected by metformin (Fig. 1C). Apoptotic cells were not observed in control or metformin-exposed hepatocytes (Fig. 1C).

Next, the effects of metformin on GCDCA-induced caspase-6 activity were studied. Metformin (1 mmol/L) decreased GCDCA-induced caspase-6 activity by 46% (Fig. 1D).

**Figure 2:**
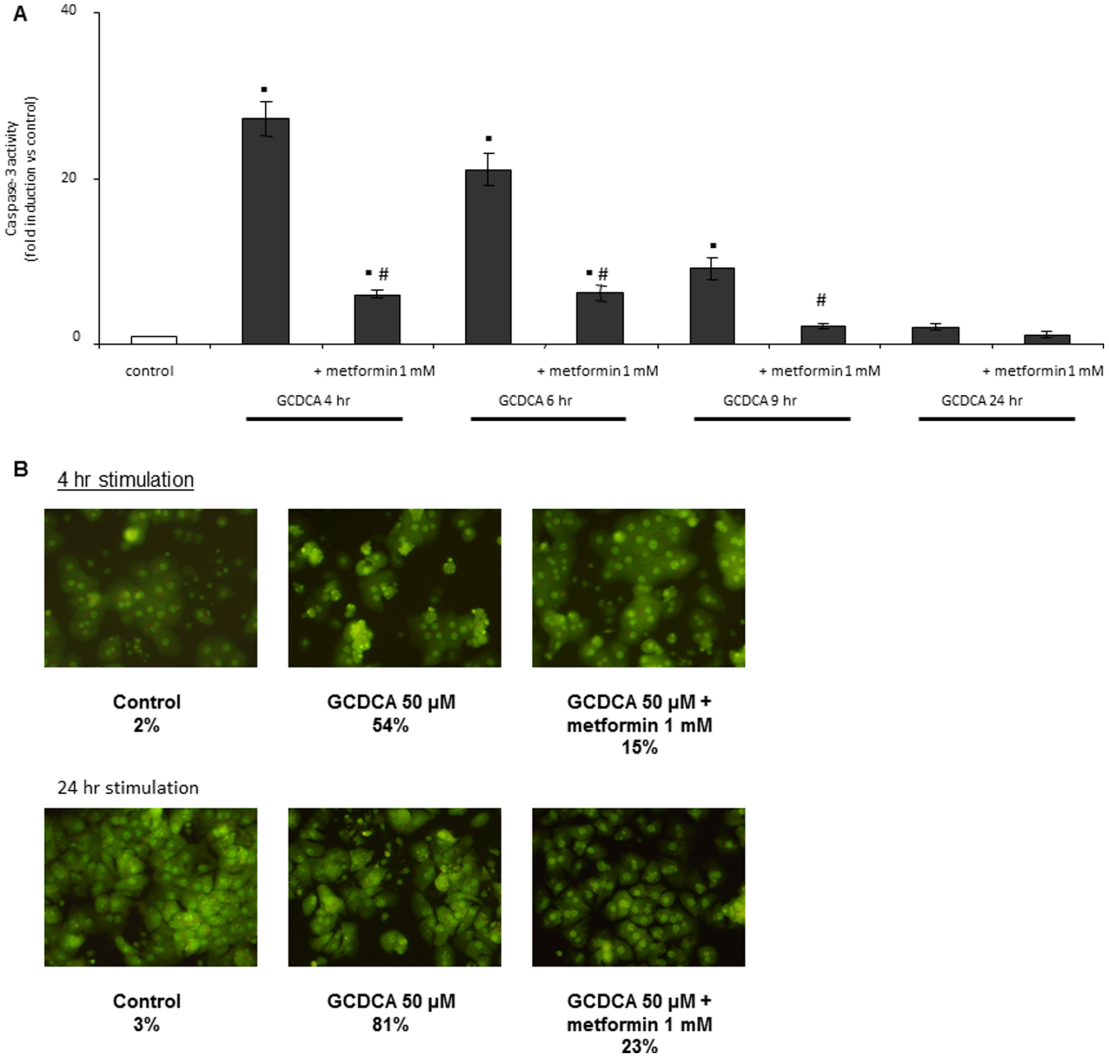
Metformin does not delay bile acid-induced apoptosis. Primary rat hepatocytes were exposed to GCDCA (50 µmol/L) without or in combination with metformin for 4, 6, 9 and 24 hrs. Metformin (1 mmol/L) was added 10 minutes before GCDCA. Control cells are rat hepatocytes not treated with GCDCA or metformin. At the indicated time points, cells were analyzed for (A) caspase-3 like activity, or (B) nuclear condensation as indicated by acridine orange staining and expressed as % condensed nuclei. Magnification 20X. N = 4 for each experiment. Statistical analysis: ▪ p<0.01 compared to control. # p<0.05 compared to GCDCA-treated cells at the corresponding time-point.

To demonstrate that metformin inhibits and not delays bile acid induced apoptosis, a time course study was performed. At the indicated time points, from four to nine hrs after addition of GCDCA, metformin significantly inhibited GCDCA-induced caspase-3 activity (Fig. 2A). After 24 hrs, the caspase-3 activity levels of GCDCA and GCDCA plus metformin treated cells were both comparable to control. Acridine orange staining was performed to ensure that the peak of caspase-3 activity of hepatocytes treated with GCDCA plus metformin was not between any of the time points analyzed. Both 4 and 24 hr after exposure, GCDCA-induced nuclear condensation was reduced by metformin by 72% (Fig. 2B).

### Metformin does not Block the Uptake of GCDCA at the Cell Membrane

**Figure 3:**
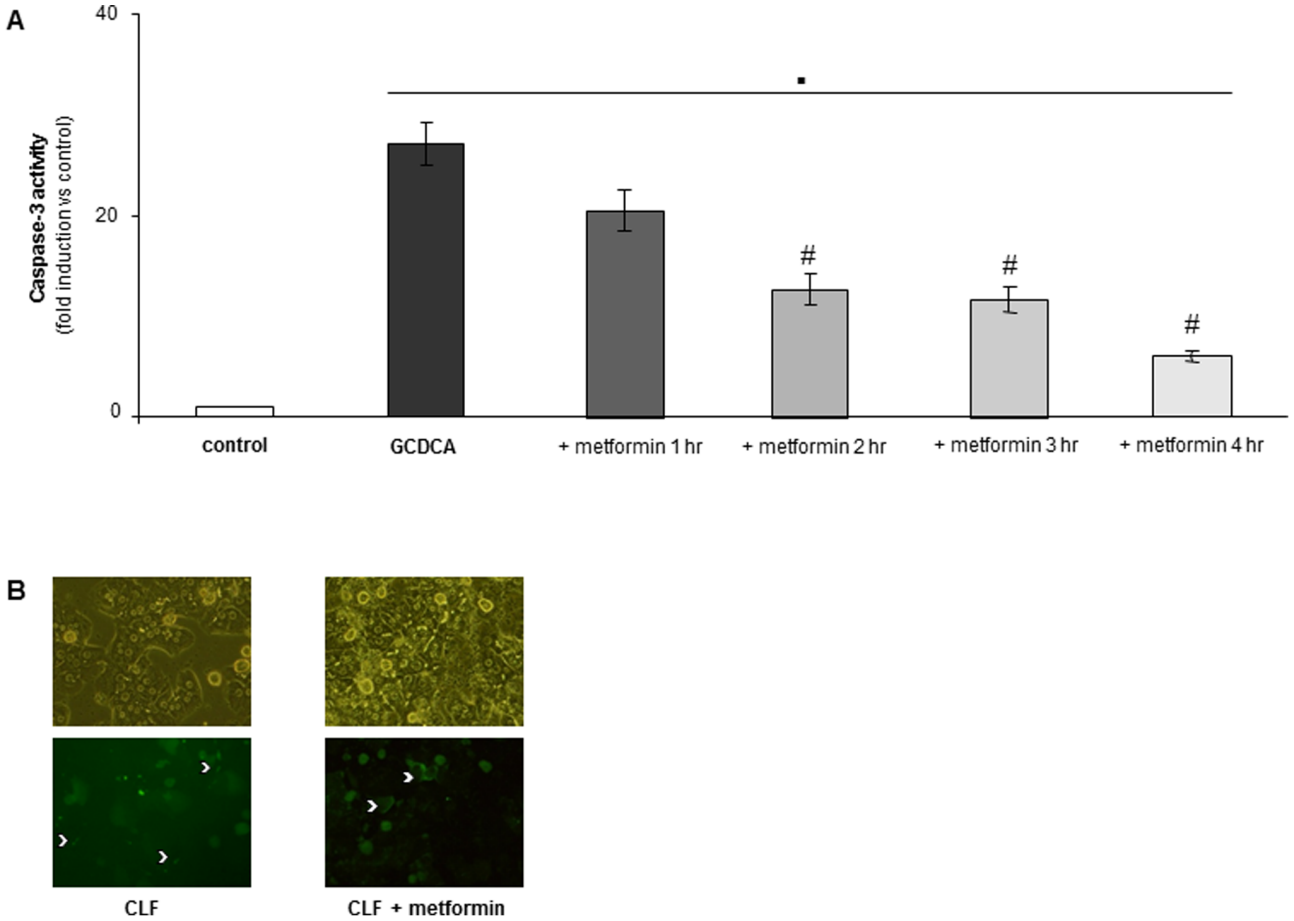
Metformin protects primary hepatocytes against apoptosis when added after GCDCA, but not by inhibiting bile acid uptake. Rat hepatocytes were treated without (control) or with GCDCA (50 µmol/L) for 4 hrs (A). GCDCA-treated hepatocytes were co-incubated with metformin (1 mmol/L) for 1–4 hrs. A longer incubation time with metformin is visualized in the graph by a color change from dark grey to white. Caspase-3 like activity was measured and is shown as fold induction compared to control values; control values were set at one. N = 4. Statistical analysis: ▪ p<0.01 compared to control. # p<0.05 compared to GCDCA-treated cells. The protective effect of metformin is not caused by reduced uptake of GCDCA (B). Primary rat hepatocytes were exposed to the fluorescent bile acid CLF (2 µmol/L) for 1 hr. Metformin (1 mmol/L) was added 10 minutes prior to CLF. Uptake of CLF by hepatocytes was visualized by fluorescent microscopy. N = 3. Representative images 1 hr after incubation are shown. White arrow: CLF uptake in the bile canaliculi. Upper panel: phase contrast. Lower panel: fluorescence. Magnification 20X.

Next, we examined whether metformin only protects cells when added prior to GCDCA, or also after bile acid exposure. Metformin was added either 10 minutes before, simultaneously with, or 1, 2 or 3 hrs after addition of the bile acid. Metformin significantly decreased GCDCA induced caspase-3 activity when added up to 2 hrs after GCDCA (Fig. 3A). No difference in reduction of caspase-3 activity between a pre-incubation of 10 minutes with metformin or addition at the same time as GCDCA was detected (data not shown). Furthermore, equal uptake of fluorescent bile acids in hepatocytes treated with or without metformin was observed (Figure 3B). These results indicate that the protective effect of metformin is not due to reduced uptake of GCDCA into the cell.

### Metformin Reduces Apoptosis without Increasing Necrotic Cell Death

**Figure 4:**
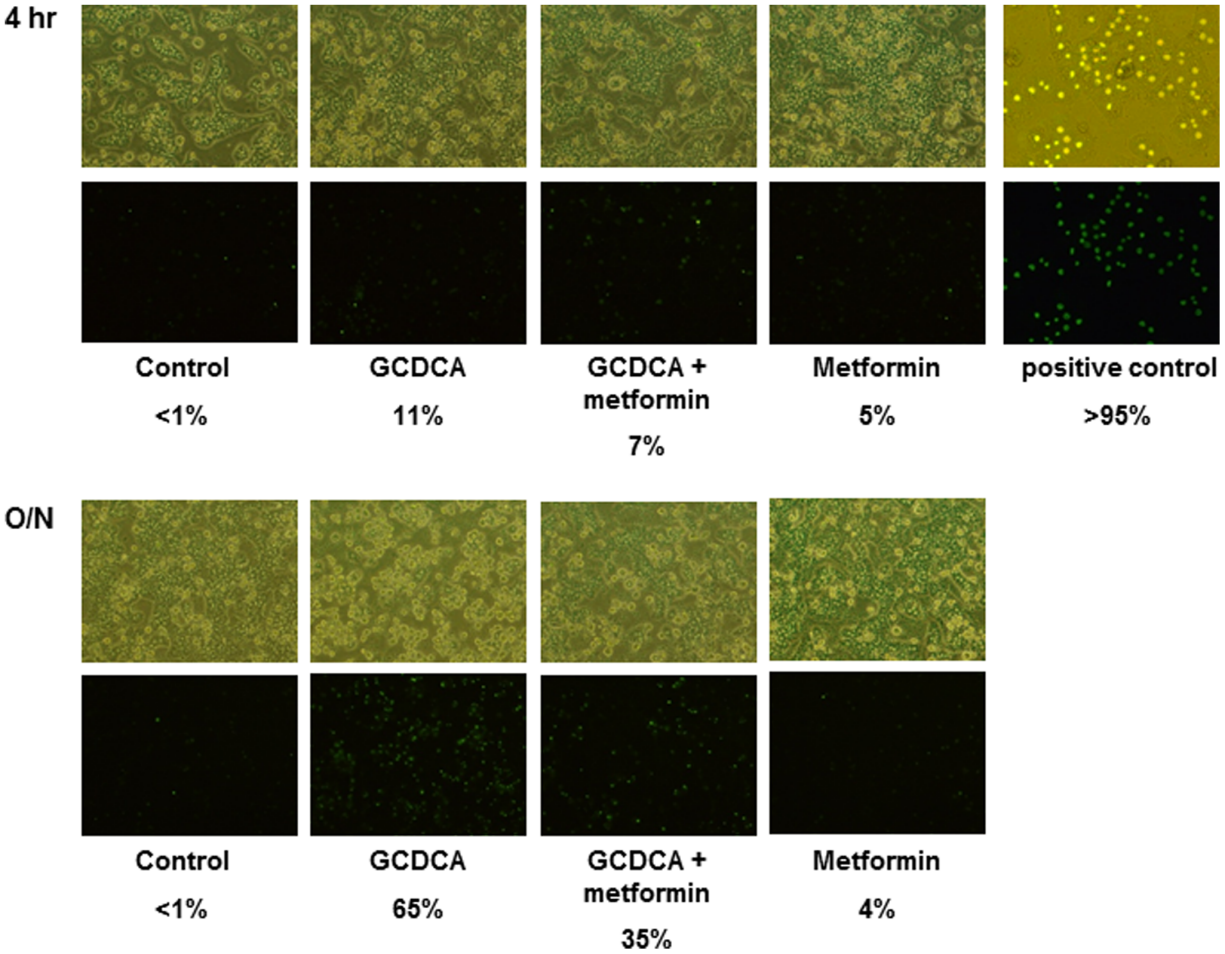
Metformin reduces apoptosis without increasing necrotic cell death. Cells were exposed to GCDCA (50 µmol/L) for 4–16 hrs. Metformin (1 mmol/L) was added 10 minutes before addition of GCDCA. Cells stimulated with H_2_O_2_ (5 mmol/L, 6 hrs) were used as positive control. Sytox green nuclear staining was used to determine necrotic cell death. N = 5. Representative images 4 hrs and 16 hrs after stimulation are shown, percentages in panels indicate % of necrotic cells. Upper panel: phase contrast. Lower panel: fluorescence. Magnification 10X.

Subsequently, we studied whether the reduction in apoptotic cell death by metformin results in a shift towards necrotic cell death. At the peak of caspase-3 activity, the percentage of necrotic cells was determined. GCDCA caused necrosis in 11% of the cells, whereas 7% of the hepatocytes exposed to GCDCA and metformin were necrotic (Fig. 4). Also after 16 hours exposure, no significant differences between cultures treated with GCDCA or GCDCA plus metformin (1 mmol/L) could be observed (Fig. 4). No significant necrosis was observed in control cultures or cultures exposed to metformin alone (Fig. 4).

### The Anti-apoptotic Effects of Metformin are Dependent on PI3K/Akt Signaling, but not on AMPK/mTOR Signal Transduction

**Figure 5:**
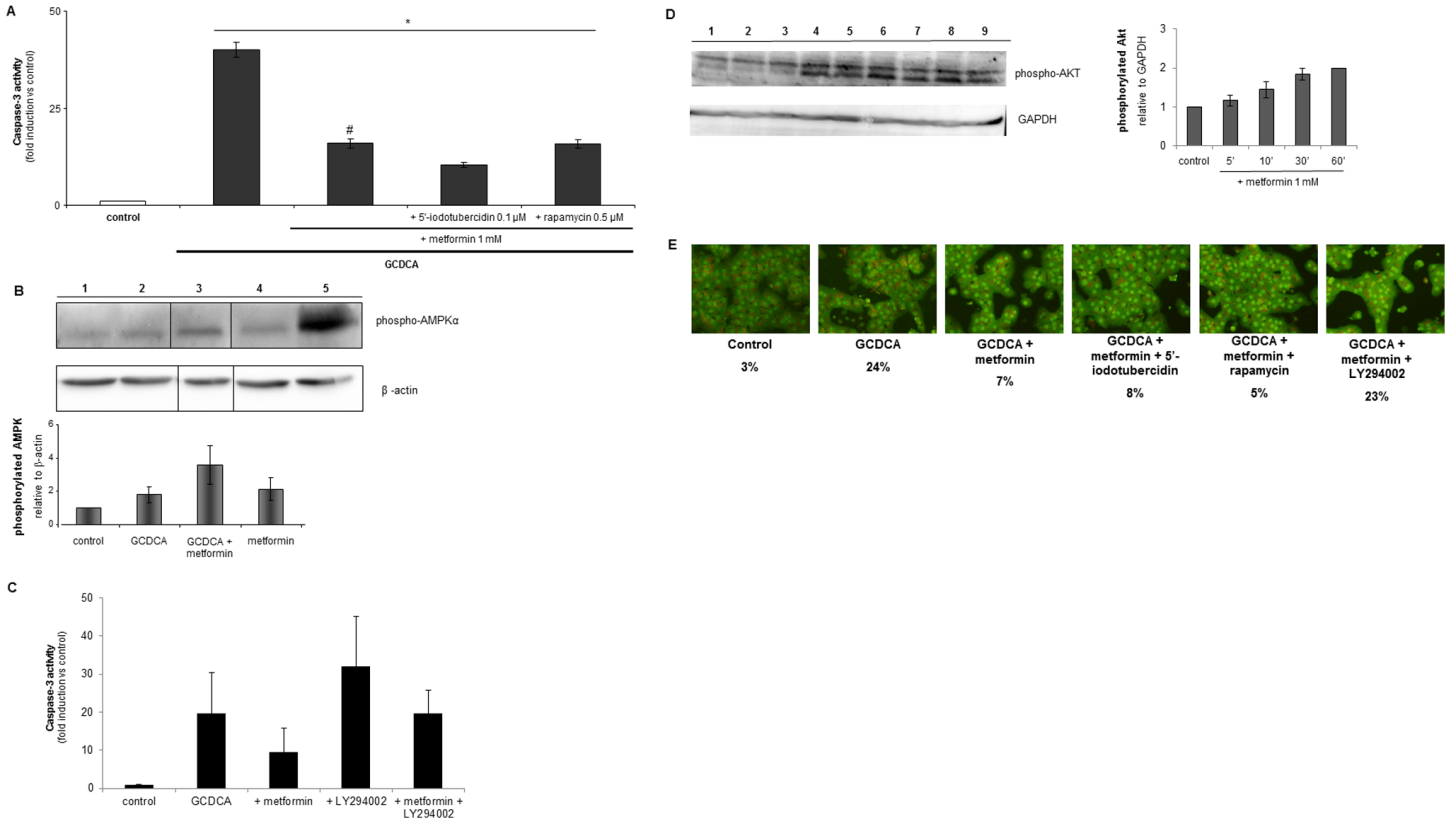
The protective effect of metformin involves the PI3-kinase/Akt pathway, but not the AMPK/mTOR pathway. (A) Primary rat hepatocytes were exposed to GCDCA (50 µmol/L) and metformin (1 mmol/L) for 4 hrs with or without 5′-iodotubercidin (0.1 µmol/L), an inhibitor of AMP-activated protein kinase, and the mTOR inhibitor rapamycin (0.5 µmol/L). Caspase-3 like activity is presented as fold induction compared to control values; control values were set at one. Data are presented as mean of at least three independent experiments +/−S.E.M. Statistical analysis: * p<0.05 compared to control. # p<0.05 compared to GCDCA-treated cells. (B) Western blot analysis for AMPK phosphorylation on cell lysates (45 µl). Lane 1: control hepatocytes; lane 2: GCDCA treated cells (50 µmol/L, 1 hr); lane 3: GCDCA plus metformin (1 mmol/L); lane 4: metformin (1 mmol/L); lane 5: positive control: hepatocytes exposed to the known AMPK activator AICAR (250 µmol/L, 1 hr). All lanes are obtained from the same membrane with the same detection reagent and exposure time. Western blots were quantified and band intensities of phosphorylated AMPKα were expressed as ratio of the intensity of β-actin protein levels. Data are presented as mean of at least three experiments +/− S.E.M. Control values were set at one. A representative Western blot is shown. (C) Caspase-3 activity of heptacoytes exposed to GCDCA (50 µmol/L) for 4 hrs and metformin (1 mmol/) with or without LY294002 (50 µmol/L). Caspase-3 like activity is presented as fold induction compared to control values. Control values were set at one. Data are presented as mean of at least three independent experiments +/− S.E.M. (D) Western blot analysis for Akt phosphorylation on cell lysates (45 µl). Left side. Lane 1: control hepatocytes; lane 2, 3: metformin (1 mmol/L) 5 minutes exposed cells; lane 4, 5: metformin 10 minutes exposed cells; lane 6, 7: metformin 30 minutes exposed cells; lane 8, 9: metformin 60 minutes exposed cells. Western blots were quantified and band intensities of phosphorylated Akt were expressed as ratio of the intensity of GAPDH protein levels (Right side). Data are presented as mean of at least three independent experiments +/− S.E.M. Control values were set at one. A representative Western blot is shown. (E) Cells were exposed to GCDCA (50 µmol/L) for 4 hrs with or without metformin (1 mmol/L), 5′-iodotubercidin (0.1 µmol/L), rapamycin (0.5 µmol/L), or LY294002 (50 µmol/L). Nuclear condensation as indicated by acridine orange staining is shown, and expressed as % condensed nuclei. Magnification 20X. Representative images are shown. N = 4 for each experiment (A–E).

The involvement of the known downstream targets of metformin, the AMPK/mTOR pathway was examined. AMPK was blocked using a pharmacological inhibitor (5′-iodotubercidin), and mTOR activation was prevented by the specific inhibitor rapamycin. As shown in figure 5A and E, blocking either AMPK or mTOR had no effect on the anti-apoptotic actions of metformin and both inhibitors did not change GCDCA-induced caspase-3 activity or nuclear condensation. Hepatocytes treated with the inhibitors alone or in combination with metformin showed caspase-3 values similar to control cells (data not shown). Interestingly, Western blot analysis demonstrated that hepatocytes treated for 1 hr with either GCDCA or metformin caused a slight induction (2-fold) of phosphorylated AMPKα compared to control cells (Fig. 5B). Cells exposed to both GCDCA and metformin showed a further 1.5-fold increase of AMPKα phosphorylation compared to control hepatocytes, while cells treated with the known AMPK activator AICAR (5-aminoimidazole-4-carboxamide-1-β-d-ribofuranoside) induced an 8-fold induction of AMPKα phosphorylation.

Previous results showed the involvement of the PI3K survival pathway in the protective effect of TUDCA against GCDCA-induced apoptosis [43]. Thus, we investigated whether the PI3K pathway could be involved in the anti-apoptotic effects of metformin, using the specific PI3K inhibitor, LY294002. Metformin reduced the GCDCA-induced caspase-3 like activity with 52% (Fig. 5C). The protective effect of metformin against apoptosis was completely abolished when the PI3K pathway was blocked (Fig. 5C, E). In addition, the protein levels of phosphorylated Akt, the downstream target of PI3K, increased in time after exposure to metformin (Fig. 5D). As described previously [43], a significant increase in GCDCA-induced apoptosis was observed when the PI3K pathway was inhibited in hepatocytes (Figure 5C).

### Metformin is not an Inhibitor of NF-κB

**Figure 6:**
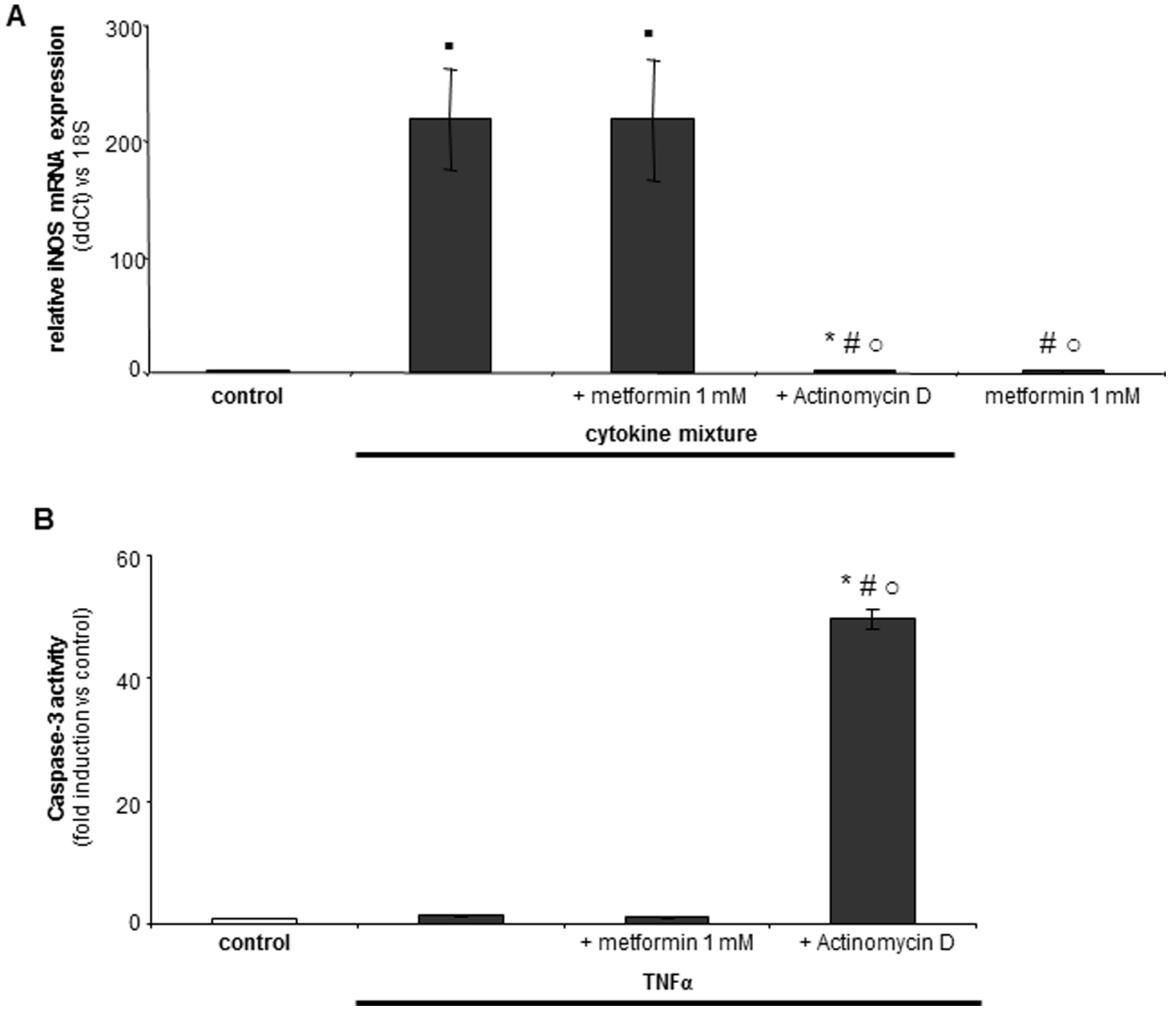
Metformin does not inhibit NF-κB activation. qPCR analysis for iNOS using cDNA of primary rat hepatocytes exposed for 4 hrs to cytokine mixture with or without 1 mmol/L metformin. The transcriptional inhibitor actinomycin D (200 ng/ml) served as positive control for NF-κB inhibition. (A). Metformin does not reduce cytokine mixture induced iNOS mRNA expression. (B) Metformin does not sensitize hepatocytes to TNFα-induced apoptosis. Caspase-3 like activity was measured in primary rat hepatocytes exposed to TNFα (20 ng/ml, 16 hrs) with or without metformin (1 mmol/L) or actinomycin D (200 ng/ml, positive control for induction of TNFα-induced caspase-3 activity). Caspase-3 activity is shown as fold induction compared to control values; control values were set at one. N = 5 for each experiment. Statistical analysis: Fig. 6A * p<0.05 or ▪ p<0.01 compared to control. # p<0.01 compared to cytokine mixture-treated cells. ○ p<0.01 compared to cytokine mixture plus metformin-treated cells. Fig. 6B * p<0.05 compared to control. # p<0.05 compared to TNFα-treated cells. ○ p<0.05 compared to TNFα plus metformin-treated cells.

Since inhibition of NF-κB sensitizes hepatocytes to TNFα-induced cell death [37], it is important to determine the effect of metformin on NF-κB activation, prior to its use in inflammatory (TNFα-mediated) liver diseases. Therefore, we studied the involvement of NF-κB signaling in the protective effects of metformin. Previous studies demonstrated strong NF-κB-dependent cytokine-induced expression of inducible nitric oxide synthase (iNOS) in hepatocytes [37]. Cytokine mixture induces a 200-fold induction of iNOS expression compared to control cells (Fig. 6A), which was not affected by metformin. As expected, the transcriptional inhibitor actinomycin D completely blocked cytokine-induced iNOS expression (Fig. 6A). Metformin alone did not induce iNOS expression and showed expression levels similar to control hepatocytes (Figure 6A). The qPCR results were confirmed using the caspase-3 assay. TNFα alone, or in combination with 1 mmol/L metformin did not induce caspase-3 activation (Fig. 6B). In contrast, caspase-3 activity was strongly induced in hepatocytes exposed to TNFα in combination with actinomycin D. Metformin exposed hepatocytes showed caspase-3 levels similar to control cells (data not shown).

## Discussion

In this study we investigated the hepatoprotective effects of metformin in models of TNFα- and bile acid-induced apoptosis in primary rat hepatocytes. Other studies described that metformin improves the viability of hepatocytes in fatty livers and reduced serum levels of ALT and AST in NASH [6]–[8]. We show that metformin is protective against bile acid-induced cell death, while it has no effect on TNFα-induced apoptosis. Metformin reduces, but does not delay, GCDCA-induced caspase activity and nuclear condensation, but has no effect on TNFα/ActD-induced hepatocyte apoptosis. The opposite effects of metformin in these two models of hepatocyte apoptosis is most likely due to the involvement of different signaling pathways in cytokine and bile acid induced apoptosis. Cytokines like TNFα exert their effects via death receptors at the cell membrane, while bile acids need to be taken up by the bile acid transporter, Ntcp [46]–[48]. Furthermore, bile acid-induced apoptosis has been demonstrated to involve activation of the Fas pathway via EGF-receptor phosphorylation [49]. TNFα is known to activate the transcription factor NF-κB, while we have previously shown that GCDCA has no effect on NF-κB signaling [42].

To elucidate the protective mechanism of metformin, we first excluded the possibility that metformin is hepatoprotective via inhibition of GCDCA-uptake at the cell membrane. The results obtained with the fluorescent bile acids clearly demonstrated that metformin has no effect on bile acid uptake. Furthermore, metformin was able to reduce apoptosis when added up to 2 hrs after bile acid exposure. These data suggest that metformin does not interfere with bile acid uptake and activates survival pathways very rapidly. In this respect, the protective effect of metformin resembles the protective effect of TUDCA, TUDCA was protective against GCDCA-induced cell death when added up to 2 hrs after the bile acid [43]. We confirm previous findings [43], that 2 hours after GCDCA exposure, caspase-3 activation is still minimal, and apoptosis can be prevented, while beyond this threshold the apoptotic machinery cannot be reversed anymore.

Previously, we demonstrated that under particular conditions the mode of cell death can shift from apoptosis to necrosis [44]. In this study, metformin does not cause a switch from apoptotic to necrotic cell death. The percentage of necrotic cells is similar for GCDCA and GCDCA plus metformin treated hepatocytes. Furthermore, metformin does not significantly reduce GCDCA-induced necrosis. These results demonstrate that metformin only protects against apoptotic cell death, but not against necrotic cell death.

Since the AMPK/mTOR pathway is an important downstream target of metformin, we investigated the involvement of these pathways in the protective effect of metformin. Our results demonstrate that the AMPK/mTOR signaling pathway is not involved in the anti-apoptotic actions of metformin [16], [17], [26], [50]. Both known inhibitors for AMPK and mTOR, 5′-iodotubercidin and rapamycin, had no effect on the reduction in GCDCA-induced apoptosis caused by metformin. Although AMPK does not appear to be involved in the protective effect, it is of interest to note that GCDCA slightly increased the phosphorylation and activation of AMPK. The mechanism of this effect and its relevance remains to be elucidated. Since AMPK is not involved in the protective actions of metformin, the drug must exert its anti-apoptotic actions via other pathways. Our results demonstrate that the PI3K/Akt pathway plays an important role in the protective effect of metformin. The protective effect of metformin against GCDCA-induced apoptosis is dependent on an intact PI3K pathway. These data are in accordance with other reports emphasizing the importance of PI3K/Akt in the protection against bile acid-induced apoptosis [34]–[36], [38], [39], [43]. On the contrary, our data imply that members of the MAP kinase family are not involved in the anti-apoptotic signaling of metformin, since inhibitors of the anti-apoptotic MAP kinases ERK and p38 do not reduce the protective effect of metformin (data not shown).

The possibility that GCDCA sensitizes hepatocytes to NF-κB activation can be excluded, since metformin, either alone or in combination with GCDCA, did not induce the expression of the NF-κB-dependent gene iNOS. One study reported caspase-regulated Bcl-xl expression, via caspases downstream of ERK1/2. Using the specific ERK1/2 inhibitor U0126, Bcl-xl expression was reduced and apoptosis was induced. This U0126-induced down-regulation of Bcl-xl was reversed by the pancaspase inhibitor Z-VAD-FMK [51]. Although a different cell type was used, caspase-dependent Bcl-xl expression could explain our results: in our model GCDCA-induced caspase activity is reduced by metformin and this could subsequently result in an additional increase in Bcl-xl expression.

Our results clearly demonstrate that metformin does not inhibit NF-κB activity. Hepatocytes are not sensitized to TNFα-induced apoptosis by metformin, and metformin does not affect cytokine induced NF-κB-dependent gene transcription. Our data are conflicting with some reports describing AMPK-mediated inhibition of NF-κB by metformin [52], [53]. However, these studies are not performed in hepatocytes but in endothelial cells, which could explain the discrepancy. Furthermore, these reports show the involvement of AMPK in metformin signaling, while in our study AMPK is not involved in the anti-apoptotic actions of metformin.

In summary, we have shown that metformin is protecting hepatocytes against GCDCA-induced apoptosis, while metformin has no effect on TNFα/ActD-induced apoptosis. The PI3K/Akt survival pathway is required for the anti-apoptotic effect of metformin; however the protection is independent of the AMPK/mTOR signaling pathway. In addition, we have demonstrated that metformin does not inhibit NF-κB activity and hence does not sensitize hepatocytes to TNFα-induced apoptosis. The latter finding would make metformin suitable in the treatment of chronic liver diseases, in which an inflammatory component is present.
